# Bisphenol A and its analogues: from their occurrence in foodstuffs marketed in Europe to improved monitoring strategies—a review of published literature from 2018 to 2023

**DOI:** 10.1007/s00204-024-03793-4

**Published:** 2024-06-12

**Authors:** Ilaria Neri, Giacomo Russo, Lucia Grumetto

**Affiliations:** 1 Department of Pharmacy, School of Medicine and Surgery, University of Naples Federico II, Via D. Montesano, 49, 80131 Naples, Italy; 2 Centre of Biomedicine and Global Health, School of Applied Sciences, Sighthill Campus, Edinburgh Napier University, 9 Sighthill Ct, Edinburgh, EH11 4BN UK; 3 Consorzio Interuniversitario INBB, Viale Medaglie d’Oro, 305, 00136 Rome, Italy

**Keywords:** Bisphenol A, Endocrine disrupting chemicals, Biomonitoring, Food safety, Food contamination, Analytical chromatography

## Abstract

In this review article, the research works covering the analytical determination of bisphenol A (BPA) and its structural analogues published from 2018 to present (February 2024) were examined. The review offers an overview of the concentration levels of these xenoestrogens in food and beverages, and discusses concerns that these may possibly pose to the human health and scrutinises, from an analytical perspective, the main biomonitoring approaches that are applied. This comes as a natural evolution of a previous review that covered the same topic but in earlier years (up to 2017). As compared to the past, while the volume of published literature on this topic has not necessarily decreased, the research studies are now much more homogeneous in terms of their geographical origin, i.e., Southern Europe (mainly Italy and Spain). For this reason, an estimated daily intake of the European population could not be calculated at this time. In terms of the analytical approaches that were applied, 67% of the research groups exploited liquid chromatography (LC), with a detection that was prevalently (71%) afforded by mass spectrometry, with over one-fourth of the research teams using fluorescence (26%) and a minority (3%) detecting the analytes with diode array detection. One-third of the groups used gas chromatography (GC)–mass spectrometry achieving comparatively superior efficiency as compared to LC. Derivatisation was performed in 59% of the GC studies to afford more symmetrical signals and enhanced sensitivity. Although the contamination levels are well below the threshold set by governments, routinely biomonitoring is encouraged because of the possible accumulation of these contaminants in the human body and of their interplay with other xenoestrogens.

## Introduction

Bisphenol A is a synthetic compound realized in the early twentieth century, emerged in the last few decades as a key monomer used as “building block” in the production of polycarbonate plastics and epoxy resins (Michałowicz 2014). The remarkable versatility, durability, and transparency of these BPA-based polymers made them the materials of choice for an array of applications, notably in the manufacturing of food and beverage packaging. As a result, the daily interaction between BPA-laden containers and the consumables they enclose has become unavoidable for billions across the globe (Usman and Ahmad 2016). BPA is found in many everyday consumer goods products made of very stable plastics, such as DVDs, plastic bowls, and thermal paper cash register receipts (Vandenberg et al. 2007), but the first evidence of its potential release from food contact material (FCM) and, of consequence, of the people exposure to this chemical due to the use of canned foods, was in 1996 by Food and Drug Administration (FDA-U.S) (Noonan et al. 2011). Produced through condensation of two parts of phenol and one part of acetone, BPA is chemically converted into a plastic polymer or epoxy resin and in the meantime firmly bonded in the polymer matrix. Unfortunately, under certain conditions, unbonded BPA residues, even if in ppm range, can be released into foodstuffs (Fattore et al. 2015; Guart et al. 2011).

The migration of BPA from packaging to food and beverages is a consequence of the dynamic interplay between the chemical composition of the packaging material and the contents it encases (Kawamura et al. 2001). This migration can be exacerbated by factors, such as temperature, storage duration and conditions, acidity, or composition of the products (Guart et al. 2011). Unfortunately, BPA can adversely interact with several hormone responsive receptors, mainly oestrogen and thyroid hormone binding, either as agonist or as antagonist, thus inducting endocrine disruption. Therefore, other bisphenol analogues, such as bisphenol S, bisphenol E, bisphenol AF, and many others, were synthesized to mitigate the endocrine-disrupting potential of BPA (Chen et al. 2016; Russo et al. 2019b). However, many BPA analogues were found to be as much or more estrogenic than BPA, as the evidence suggests that the chemical moieties responsible for the toxicological effects are those that indeed undergo the polymerisation reaction (Rosenmai et al. 2014).

For most BP analogues, the accidental release from the packaging is allowed, albeit within precisely prescribed limits. For this reason, food and beverage surveillance is crucial to assess that these limits are not exceeded. Typically, several analytical techniques such as gas and liquid chromatography with ultraviolet, fluorescence, and mass spectrometry-based detection are exploited. The present review aims at offering an overview of the analytical approaches more often applied to determine the occurrence of BPA and its analogues and their concentration levels in canned or not canned foodstuff marketed in Europe therein.

## Inclusion criteria

The eligible studies investigate the occurrence of BPA starting from 2018 to February 2024 and therefore included in the current review research. Only manuscripts in English were considered. The search was performed in different database: PubMed, Sciences Direct, Web of Science, Google Scholar, and Scopus using keywords with the following terms; “bisphenol” AND (“food” OR “vegetable” OR “vegetables” OR “fruit” OR “fruits” OR “salt” OR “sugar” OR “honey”), “bisphenol” AND “seafood”, “bisphenol” AND (“beverages” OR “milk” OR “wine” OR “beer” OR “alcohol” OR “soft drinks” OR “tea” OR “coffee”), “bisphenol” AND (“drinking water” OR “bottled water” OR “mineral water”), “bisphenol” AND (“meat” OR “poultry”), “bisphenol” AND (“cereals” OR “wheat” OR “pasta” OR “noodle”), “bisphenol” AND (“mixed food”), respectively. The output data were filtered by published year, document type, source type, and language as described above. Only primary, peer-reviewed articles were collected, whereas reviews and conferences proceedings were excluded. The inclusion criteria for this study were: all peer-reviewed research articles investigating the occurrence of BPA into foodstuffs. In the review process, articles obtained from databases are assessed based on their titles and abstracts. All the information, such as publication year, foodstuff category and subcategory, limits of detection (LOD) and limits of quantification (LOQ), separation and detection methods, and range of concentrations retrieved, were extracted and pooled in the Tables. BPs occurrence, and key findings were extracted from the articles.

## Migration of BPA and its analogues from food contact materials

From the plastics or can inner coating, BPA is released through chemical process of hydrolysis from the bonded polymer (Vom Saal and Hughes 2005) and its amount depends on temperature and/or heating duration, repeated use, brushing, type of food and packaging, manufacturing processes, irradiation, water hardness, as well as pH which could facilitate the BPA release and migration into foodstuff (Lim et al. 2009). Manufacturers are under obligation to provide information about the cleaning, sterilization methods, and product conditions on the labels of bottles or packaging. Several and various packaging materials are available in the foodstuffs industry field. These are polycarbonate (PC), polyethylene terephthalate (PET), high‐ and low‐density polyethylene (HDPE, LDPE), metallic cans (MC), polystyrene, polypropylene (PP), and polyvinyl chloride (PVC). Several studies demonstrated the BPA releasing from these materials, even if these are labelled as BPA-free, as in the case of brushed baby bottles (Ali et al. 2018). Different packaging materials were studied for BPA migration into food by the use of simulants: amounts of BPA (PC: 1.4- 35.3 mg/kg) (Ehlert et al. 2008), (HDPE caps: 0.145 μg/dm^2^) (Guart et al. 2011), (LDPE: 0.128 μg/dm^2^) (Guart et al. 2011) or below the specific migration limit set for food at 0.05 mg Kg^−1^ (Krivohlavek et al. 2023) were found. Metallic cans have on their inner surface a thin layer of epoxy resins or organosols. If the polymerization is not fully completed or if there are issues during the food canning process, BPA leaches into the food. For instance, in a recent study, it has been demonstrated the occurrence of BPA concentration ranging from 8.91 to 14.01 pg/mL in canned soft drinks (Kumar et al. 2023). Hananeh et al. investigated the BPA migration from other kind of plastics, such as PP, and they found that some BPA migrated from the materials such as stainless steel, aluminum, and silicone water bottles albeit under standard limits (Astolfi et al. 2021; Hananeh et al. 2021).

## Bisphenols regulations

**Fig. 1:**
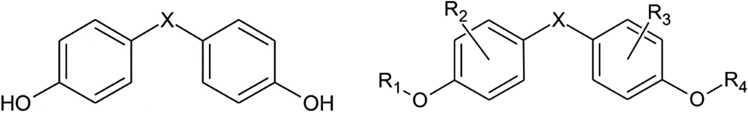
**a** Bisphenol generic structure, and **b** structure of bisphenol generic derivatives; “X” represents a portion of the molecule that can be variously replaced with additional bonded groups; "Rn" indicates potential additional or replacement groups

The EU Chemicals Regulation (EC) No 1907/2006 of the European Parliament and of the Council of 18 December 2006 concerning the Registration, Evaluation, Authorisation and Restriction of Chemicals (REACH) poses a responsibility on manufacturers and importers of chemicals to assess the potential risks posed to humans and the environment by all planned uses of BPA. They are required to complete and report their risk assessments in a chemical safety report, where the companies must outline the specific conditions under which BPA can be safely utilized throughout its entire life cycle. Additionally, they must implement the measures aimed at reducing the risks of contamination. As for the plastic products intended for food contact, the Regulation (EU) no. 10/2011 imposes a specific migration limit into foodstuffs of 0.05 mg kg^−1^ for any chemical compound. BPA is a substance that may be carcinogenic, mutagenic, or toxic for reproduction (CMR), persistent, bio-accumulative and toxic (PBT), or very persistent and very bio-accumulative (vPBT). Due to its nature, notification requirements are needed under REACH, for products that contain BPA, for its potential to cause harm to the human health. On 8 July 2021, BPA has been included in the Candidate List of Substances of Very High Concern (SVHC) under REACH specifically under Category 1B, due to its toxicity as endocrine disruptor (Agencies 2023). If the concentration of BPA in EU for sale imported products exceeds 0.1% by weight, importers and manufacturers must submit notification to European Chemicals Agency (ECHA) via the “Substances of Concern” (SCIP database) under the Waste Framework Directive (WFD). The aim is to provide as much information as possible for consumers and waste operators. BPA is restricted under various regulations in the EU regarding, specifically, consumer products, FCM, and toys. Among the various European countries, some developed their own national frameworks regulating the uses and applications of BPA, such as France, that banned its usage in all food packaging including containers and utensils, as well as teethers and soother shields to be used by consumers of all ages. Indeed, the use of BPA was originally banned in the manufacture of baby bottles, import, export, and placement on the market of France under the Law 2010-729 of 30 June 2010, the limits for the global leaching into food from plastic FCM, varnishes, and coatings were set to 0.05 mg kg^−1^. Subsequently, the Law No. 2012-1442 of 24 December 2012 expanded the restriction including all the packaging, container, or utensil intended to come into direct contact with food ((EU) 2011). Nevertheless, the import and sale of BPA-containing products for food contact purposes remain prohibited in France, and the French Constitutional Council ruled out that this ban does not apply to the marketing of such products in other countries. Since 2010, Denmark banned BPA in FCM for children under 3 years and included it in the Danish Environmental Protection Agency's List of Undesirable Substances (LOUS) (Agency 2022). In 2013, Belgium and Sweden banned the use of BPA in FCM intended for children less than 3 years old and in plastic articles like spoons and plates, as well as the Austrian law prohibited to manufacture pacifiers and teething rings with BPA (2011). In 2016, Sweden banned the use of BPA in the epoxy resins lining water pipes, but this legislative provision was withdrawn in the same year (Federal Law Gazette Part II 2011). Furthermore, in 2022, the German Federal Office for Chemicals (BfC) submitted a dossier including a proposal to ECHA to restrict the use of BPs. Successively, the dossier has been temporary withdrawn, since the German authorities advised that a significant proposal re-drafting was necessary and beneficial to achieve the intended goals. The updated Drinking Water Directive (EU) 2020/2184 requires water intended for human consumption to have a maximum of 2.5 μg L^−1^ of BPA and the Member States must comply with these standards by 12 January 2026. This directive also extend to the testing of polymeric materials in contact with drinking water, to ensure that they do not cause any BPA migration into water. Concerning the threshold of the tolerable daily intake (TDI) for humans, the European Food Safety Authority (EFSA) published in 2006 its first BPA-related risk assessment, setting a threshold of 50 µg kg^−1^ body weight/day. In 2015, an updated assessment of exposure to BPA and its toxicity determined the lowering of this threshold to 4 µg kg^−1^ body weight/day (EFSA Panel on Food Contact Materials and Aids 2015). In 2023, EFSA published a scientific opinion on the de novo evaluation of public health risks related to the presence of BPA in foods, with TDI threshold lessened to 0.2 ng kg^−1^ body weight/day (Ramírez et al. 2023). As a consequence of BPA regulatory actions, industries shifted toward use of BPA alternatives or “BPA-free” products. Figure 1 represents the generic chemical structures of a bisphenol and bisphenol derivative.

It can be assumed that, if a molecule has a chemical structure close to that of BPA, it may also share some of its toxicity. Understanding the potential risks of BPA substitutes is important to ensure the continued protection for human health and environment. Within the EU, three bisphenols (BPA, bisphenol B (BPB), and 2,2-bis(4′-hydroxyphenyl)-4-methylpentane) (BPP) have been identified as SVHCs. BPB as endocrine disrupting chemical shows adverse effects on the male reproductive system in rodents and fishes, clear estrogenic effects in rats and fishes, and possibly anti-androgenic effects, giving rise to an equivalent level of concern to those of other substances listed in points (a) to (e) of Article 57 of the REACH Regulation (2022) (Serra et al. 2019). ECHA gathered and catalogued bisphenol derivatives obtaining a total of 148 substances including 17 “bisphenols” with the generic “bisphenol” structure, and “bisphenol derivatives” having common structural features. This list was published in December 2021 in “Assessment of regulatory needs-Group Name: Bisphenols” (2022).

According to ECHA opinion, 34 BPs of this group should be restricted under the EU’s chemicals legislation REACH as these may interfere with hormonal systems, even if the number may change after the acquisition of new data. However, a group of 26 suspected BPs may still be regulated in consumer goods due to their skin sensitisers action. Germany, that has proposed to restrict BPA in products to 0.001% by weight to reduce the amount of endocrine-disrupting chemicals released into the environment, has extended this rule also to other BPs, such as Bisphenol B, S, F, and AF. The European commission set a migration limit for certain epoxy derivatives in material and articles intended to come into contact with foodstuffs and food simulants up to 9 mg kg^−1^ and 9 mg dm^−2^ respectively, for the sum of BADGE (CAS No [1675-54-3]) and its adducts with water (BADGE.H_2_O [76002-91-0] and BADGE.2H_2_O [5581-32-8]) and up to 1 mg kg^−1^ and 1 mg dm^−2^, respectively, for the sum of BADGE and its adducts with hydrochloric acid (HCl) (BADGE.HCl [13836-48-1], BADGE.2HCl [4809-35-2] & BADGE.H_2_O.HCl [227947-06-0]) ((EU) 2005). Instead, Bisphenol F diglycidyl ether (BFDGE) CAS No [39817-09-9] has been prohibited since 2005 ((EU) 2005).

## Toxicological aspects and human health effects

Exposure to BPA has gained considerable global health attention due to its potential adverse health effects on human health. Depending on exposure extent, several studies indicated a relationship between BPA exposure and negative health outcomes (Liao and Kannan 2012). Bisphenol A has been linked to various human health hazards, mostly related to reproductive, metabolic, and immune system disorders. Indeed, many studies on humans showed that the most noticeable effect is its disruption of sex hormone activity, with direct influence on the development and function of the reproductive system, both on males and f﻿em﻿ales.

Additionally, BPA causes alteration in thyroid function, stimulating the pituitary gland to produce thyroid hormones. These hormones regulate many activities in the body such as glucidic/lipidic metabolism and heartbeats. Indeed, the exposure to BPA affects the cardiovascular system (Rochester 2013) as well as may contribute to the development of hormone-dependent pathologies like obesity, or type 2 diabetes (Molina-López et al. 2023). Furthermore, due to its xenoestrogen properties, it has been linked to various types of cancer, such as liver, gastrointestinal, breast, and skin tumours (Dueñas-Moreno et al. 2023). To date, a substantial amount of literature suggests that maternal BPA exposure can have a negative impact on the outcomes of offspring in developing systems; for instance, it increases the risk of behavioural problems, associated with hyperactivity disorder, antisocial behaviour, sleep related issues, and language development (Jensen et al. 2019; Kanlayaprasit et al. 2021). Primary route of BPA is through the diet, i.e., canned foods and water bottles, secondary routes include inhalation, and transdermal intake. After ingestion, BPA is rapidly absorbed in the bowel as monoester forms due to their rapid hydrolysis by the gut lipases or esterase. As part of the phase II metabolism, BPA is converted into namely BPA glucuronide and BPA sulphate within the gastrointestinal tract and liver of humans and eliminated through urine, faeces, and sweat (Andra et al. 2016). The bioavailability of BPA is dependent on the exposure route and, therefore, it is an important factor for assessing BPA exposure risks in humans. BPA can be measured in serum as well as urine. Urine BPA testing is an alternative to more invasive methods; however, because of the rapid metabolism and excretion of BPA (Dekant and Völkel 2008), it merely measures excreted BPA and does not take into account current in vivo exposure (Dekant and Völkel 2008).  Thus, serum may provide a more accurate and realistic indicator of exposure.

## Analytical method for BPs analysis

Food and beverages can contain BPA and/or its analogues in both fresh and canned commodities because of migration from the food contact material or due to the previous contamination in the food chain (Russo et al. 2018, 2019a, b). Sample preparation is particularly crucial in food analysis as it allows signal suppression of the matrix, enhanced sensitivity, and concentration of the target analytes, thereby allowing trace analysis.

Tables 1 and 2 summarise the main sample preparation approaches. Solid food is typically subjected to homogenization, while liquid samples undergo degassing, filtration, and/or centrifugation. Samples with high protein content may require protein removal through precipitation, which can be acid, salt, or solvent mediated (Polson et al. 2003). Drinking waters are added of ascorbic acid to remove the excessive residual chlorine by products (Petraccia et al. 2006). Canned foods that contain both liquid and solid components are usually filtered and treated separately.

**Table 1:** Liquid chromatographic methods for BPs determination

Y 20	Matrix	PKG	Analytes	Sample amount extraction	Separation (column, elution) Detection	LOD and LOQ	% Recovery	Ref
18	Fish	NC	BPA	0.5 g QuEChERS Kit ACN/H_2_O 4:3	Waters Acquity BEH C18 H_2_O/MeOH pH 9 Gradient MS	0.003 and 0.008 ngg^−1^	55.6–109.9	Pico et al. (2019)
18	Mixed	C + Pp	BPA + 16 6 BDGEs	1 g SPE (Oasis HLB)	Waters Acquity BEH C18 H_2_O/ACN 90:10 (v/v) MS	< 0.0007 and 0.0108 ng mL^−1^	90–104	van Leeuwen et al. (2019)
18	Vegetables	NS	BPA	0.2 g *d*-SPE (Alumina, Florisil and silica sorbents)	HALO C18 H_2_O/MeOH Gradient MS	0.025 and 0.167 ng g^−1^	94–102	Aparicio et al. (2018)
18	Milk and Dairy (Infant formula)	NS	Tetrabromobisphenol	1 g DLLME (MeCN, MgSO4 and NaCl in acidic conditions)	Kinetex C18 MeOH/ H_2_O 90/10 (v/v) MS	0.04 and 1 ng g^−1^	88.5	Martinez et al. (2019)
18	Beverages	NS	BPA	0.5 L SPE (Oasis HLB)	Hypersil Gold H_2_O/ACN Gradient MS	0.025 and 0.05 ng g^−1^	101.8	Huysman et al. (2019)
18	Raw and Cooked Seafood	NC	BPA	1 g Pressurized liquid extraction with MeOH + gel permeation chromatography	Acquity BEH C18 H_2_O/MeOH pH 9 Gradient MS	0.03 and 0.20 ng g^−1^	62.6–109.9	Alvarez-Munoz et al. (2018), Jakimska et al. (2013)
18	Milk and Dairy, Raw Milk	NC	BPA	2.5 mL SPE (Chromabond C18)	Synergi Fusion-RP 80 Å, ACN/H_2_O 70:30 (v/v) FLD	0.01 and 0.03 ng g^−1^	np	Santonicola et al. (2018)
18	Vegetable	NC	BPA and BPF	1.0 g matrix solid-phase dispersion	Ascentis Express RP-Amide H_2_O/ACN Gradient MS	240 and 0.77 ng g^−1^	38	Margenat et al. (2018)^b^
19	Beverages	C	BPA + 6	20 mL Strata TMX	Supelco C18 ACN/H_2_O 55:45 FLD	2.06–21.37 ng mL^−1^	70.2–106.7	Russo et al. (2019b)
19	Beverages	C	BPA, BPB, BPF BADGE BFDGE	5 mL BPA Affinimip® SPE	AscentisExpressRP-Amide H_2_O/ACN Gradient FLD	0.15/0.50 ng mL^−1^	76–103	Cirillo et al. (2019), Gallo et al. (2017)
19	Milk and Dairy	NC	BPA	2.5 mL SPE (Chromabond C18)	Synergi Fusion-RP 80 Å, ACN/H_2_O 70:30 FLD	0.005–0.016 ng g^−1^	70–100	Santonicola et al. (2019)
19	Fish	NC	BPA	0.5 g QuEChERS,( PSA, C18; GCB, graphitized carbon black, MgSO_4_)	Acquity BEH C18 H_2_O/MeOH pH 9 Gradient MS	5 × 10^–4^ -15 ng g^−1^	53–102	Jakimska et al. (2013), Pico et al. (2019)
19	Beverages	C	BPA, BPB,BPF	5.0 mL SPE (Strata® C18)	Kinetex® PFP H_2_O/MEOH Gradient MS	0.15 and 1.0 ng mL^−1^	74–98	Gallo et al. (2019)
19	Mixed	P, G, C, Pp	BPA + 12	SPE (DSC-18) LLE (ACN/n-pentane)	Kinetex Phenyl-hexyl H_2_O/MEOH Gradient MS	n.p. and −85 ng g^−1^	37 –120	Vavrouš et al. (2019)
20	Mixed	C	BPA	All cans content MAE-MISPE polypropylene cartridges	Discovery C18 RP H_2_O/MeOH (70/30 v/v) MS	0.9 and 4.6 ng g^−1^	51–57	Maragou et al. (2020)
20	Mixed	C	BADGE + 3 BFDGE + 3	0.1 g Ultrasound-assisted solvent extraction of porous membrane	Kinetex® XB-C8 column Ammonium formate/MeOH Gradient MS	0.27 and 0.49 ng g^−1^ 0.78 and 1.5 ng g^−1^	78.3–112.6	Szczepańska et al. (2020)
20	Fish	NP	BPA	2.00 ± 0.01 g AFFINIMIP® SPE	Kinetex® PFP H_2_O/MeOH Gradient MS	0.15 and 0.5 ng g^−1^	101.1	Di Marco Pisciottano et al. (2020)
20	Vegetables	C	BPA	SPME	Luna® C18 H_2_O/ACN Gradient FLD + MS	5 and 10 ng g^−1^	72–90	Vilarinho et al. (2020)
21	Mixed food and beverages	C	BPA + 19	Whole content of canned food/1500 uL SUPRAS hexanol/THF/water hexanol/ THF	ACE 3 C18-PFP H_2_O/MeOH Gradient MS	n.p and 0.06–0.81 ng g^−1^ n.p. and 0.019–0.19 ng g^−1^	73–114	Caballero-Casero and Rubio (2021)
21	Mixed and infant food	G,C,Pp,P	BPA + 7	5.0 ± 0.05 g for milks, fruit mixes and vegetable mixes 2.0 ± 0.02 g of infant cereals Liquid extraction (MeOH or MeOH/acetic acid) -SPE online	C18 column H_2_O/ACN Gradient MS	0.2–2 and 1–10 ng g^−1^	np	Sirot et al. (2021)
21	Mixed	P, C, Pp	BPA + 6	2 g Liquid extraction (ACN/H_2_O)	Acquity UPLC® BEH C18 column H_2_O/MeOH Gradient MS	0.1–1 ng/g and 0.4–4.0 ng g^−1^	91–105	Gálvez-Ontiveros et al. (2021)
21	Milk	C + P + Pp	BPA	2 g Matrix solid-phase dispersion (MSPD) Florisil®	Acquity UPLC® BEH Phenyl column H_2_O/ACN Gradient MS	0.00097 and 0.0032 ng g^−1^	np	Herrero et al. (2021)
21	Beverages	C	BPA + 12	5 g Heptane solution and ACN	Phenomenex® Phenosphere ODS columns H_2_O/ACN:MeOH Gradient FLD	5 and 12.5 ng g^−1^	75–102	Lestido-Cardama et al. (2021)
21	Milk	TP (Pure-Pak®)	BPA and BPF	1 mL Chromabond C18 SPE	Synergy Fusion-RP column ACN/H_2_O (70:30, v/v) FLD	0.03 and 0.1 ng mL^−1^	78.4–107.2 (BPA) 97.60–107.16 (BPF)	Mercogliano et al. (2021), Santonicola et al. (2021a)
21	Seafood	NP	BPA, BPF, BPS	0.1 g Matrix solid-phase dispersion-Florisil®	Symta ACE 5 C18-PFP column H_2_O/ACN Gradient DAD	0.07–0.29 ng g^−1^ 0.25–1.12 ng g^−1^	78.5–87.0	Cañadas et al. (2021)
22	Beverages	C	BPA, BPF,BPAF	10 mL VA- DLLME MeOH/, octanoic acid 1:1 pH 6.00	BEH C 18 H_2_O/MeOH Gradient MS	0,045–9.45 ng mL^−1^	70–120	Baute-Pérez et al. (2022)
22	Fish	C	BADGE, BADGE·H_2_O BADGE·HCl, BADGE·2H2O BADGE·2HCl BADGE·H_2_O·HCl BFDGE	10 g QuEChERS The dispersive kit used for dispersive solid-phase extraction (dSPE) contained 150 mg magnesium sulfate, 25 mg primary–secondary amine (PSA), and 25 mg C18	Poroshell 120 SB-C18 H_2_O /MeOH Gradient MS	0.2 and 5.2 ng g^−1^	85–105	Lapviboonsuk and Leepipatpiboon (2014), Toptancı et al. (2022)
22	Dairy and mixed	NC	BPA + 8	5 g/3 g ACN;QuEChERS (MgSO4, NaCl,NaOAc)	Agilent Zorbax SB-C18 H_2_O/ACN Gradient MS	0.30 and 1.5 ng g^−1^	np	Liotta et al. (2022), Xiong et al. (2018)
22	Beverages	C	BPA,BPB,BPS	2 mL Strata X-PRO	Luna Polar H_2_O/MeOH Gradient MS	0.26–0.78 ng mL^−1^	78–105	Schiano et al. (2022)
22	Beverages Milk	NC	BPA + 7 BADGE BFDGE	1 mL LLE 5 mL AFFINIMIP	Kinetex Phenyl-Hexyl H_2_O/MeOH Gradient MS	0.003–0.01 ng mL^−1^ 0.15–5 ng mL^−1^	64.2–106.3	Di Marco Pisciottano et al. (2022a)
22	Milk	NC + Pp + P	BPA, BPS, BPF, BPAF, BPB, BPE	1 mL QuEChERS (acetic buffer/ACN/NaClMgSO4)	Hypersil Gold C18 ammonium acetate/ACN Gradient MS	0.19 ng mL^−1^ and n.p	> 80	Frankowski et al. (2022)
22	Beverages Milk	NC + TP + P	BPA + 19	1 mL LLE 5 mL AFFINIMIP	polar-embeddedC18 H_2_O/ACN Gradient FLD MS	0.2–3 ng mL^−1^ W 0.003–1 ng mL^−1^ M 0.03–5 ng mL^−1^ –1 ng mL^−1^	np	Di Marco Pisciottano et al. (2022b)
23	Mixed	NS	BPA, BADGE + 7 derivatives	1 g SPE (ENVI- Carb®) and QuEChERS	Raptor Biphenyl H_2_O/ACN Gradient MS	0.2–0.6 ng g^−1^	85–107	Toptancı (2023)

*NS* Not Specified, *NP* Not Packed, *TP* Tetrapack, *C* Canned, *NC* Not Canned, *G* Glass, *P* Plastic, *Pp* Paper, *np* not present

**Table 2:** Gas chromatographic methods for BPs determination

Y 20	Matrix	PKG	Analytes	Sample amount extraction	Separation (column) detection	Derivatisation (technique)	LOD and LOQ	% Recovery	Ref
18	Ready to eat baby food	P	BPA + 6	2.0 g QuenChers (Dispersive PSA solvent)	ZB-5MS capillary column MS	Yes (Silylation)	0.1/1 ng g^−1^ and 0.5/4 ng g^−1^	91–110	Garcia-Corcoles et al. (2018)
18	Herbs and spices	NP	BPA	0.5 g QuenChers (Dispersive PSA solvent, MgSO_4_ and PSA)	Supelco SPB-5MS MS	No	1.303 and 3.013 ng g^−1^	83–110	Di Bella et al. (2018)
18	Vegetable	NC	BPA andBPF	5.0 g matrix solid-phase dispersion	Sapiens X5-MS MS	Yes, (methylation of the acidic carboxylic groups)	NP	38	Margenat et al. (2018)^b^
18	Milk and Dairy, Infant formula	NS	BPA	1 g of homogenised sample DLLME 20 µL of HCl 3 N until pH < 5 followed by 2.5 mL of MeCN, 1 g of anhydrous MgSO4 and 0.25 g of NaCl, shake vigorously by hand for 5 and centrifuge the tube	DB-5MS column MS	No	0.02 and 0.05 ng mL^−1^	88	Martinez et al. (2019)
19	Red wine	NC	BPA	10 mL CH2Cl2/NaOH USVADLLME	VF-1 ms Varian MS	No	1.05 and 5.7 ng mL^−1^	85–95	Cinelli et al. (2019)
19	Milk	C	BPA	2 g DLLME	DB-5MS MS	Yes, acetylation	0.02 and 0.05 ng mL^−1^	88^a^	Cunha et al. (2011), Martinez et al. (2019)
20	Spices and aromatic herbs	NC	BPA + 18	SPE (Cao et al. 2015)	Supelco SPB-5MS MS	No	0.005 and 3.013 ng g^−1^	83–110	Lo Turco et al. (2020)
20	Honey	NS	BPA	2.5 g UVA-DLLME	Fused silica TRB-5MS MS	No	11 and 22 ng g^−1^	71.5–100.4	Notardonato et al. (2020)
20	Mixed	C and NC	BPA + 8	DLLME tetrachloroethylene and acetic anhydride	DB-5MS column MS	No	np	np	González et al. (2020)
20	Meat	C	BPA + 8	5.0 g QuEChERS-DLLME tetrachloroethylene and acetic anhydride	DB-5MS column MS	Yes, acetylation	0.15 and 0.5 ng g^−1^	67–101	Cunha et al. (2020)
20	Cereal grains	NS	BPA + 1	2.0 g UAE with AcEt:MeOH (90:10 v/v) containing 3% of NH4OH -dSPE with PSA	Agilent HP-5MS column MS	Yes, silylation	0.05–0.4 and 0.1–1.2 ng g^−1^	30–119	Albero et al. (2020)
20	Fish	NS	BPA + 6	1 g muscle liver DLLME with tetrachloroethylene and acetic anhydride	DB-5MS column MS	Yes, silylation	0.9 and 1.3 ng g^−1^	63–106	Barboza et al. (2020)
20	Cereal-based foodstuff	NS	BPA	2.0 g Polymeric sorbent LiChrolut EN and Oasis HLB (UAE-ASPE)	DB-5MS fused silica capillary column MS	Yes, silylation	0.006 ng g^−1^ and n.p	82–105	Azzouz et al. (2020)
21	Vegetables and fruits	NS	BPA	2.0 g UAE-SPE (hydrophilic Millex-LG PTFE)	DB-5MS column MS	Yes, silylation	0.006–0.025 ng g^−1^	83–110	Hejji et al. (2021)
21	Honey	P, G	BPA	1 g DLLME (ACN and chloroform)	Agilent HP-5MS column MS	No	0,6 and 2.0 ng g^−1^	82	Peñalver et al. (2021)
22	Fish	NC	BPA + 6	0.5 g QuEChERS (NaCl, NaSO4,dSPE EMR-Lipid sorbet) (DLLME (ACN/AA/CCl4	ZB-XLB MS	4,4-DDT-d8/tonalide-d3	0.5 and 100 ng g^−1^	70–120	Petrarca et al. (2022a, 2022b)
23	Food and beverages	NS	BPA + 15	10 g QuEChERS (magnesium sulfate) method	OPTIMA-5 MS column MS	Yes, with bis(trimethylsilyl)trifluoroacetamide (BSTFA)	0.03–1.66 and 0.10–5.55 ng mL^−1^	25–120	Lucarini et al. (2023)

*NS* Not Specified, *NP* Not Packed, *TP* Tetrapack, *C* Canned, *NC* Not Canned, *G* Glass, *P* Plastic, *Pp* Paper, *np* not present

^a^Average value

^b^GC–MS/MS for qualitative analysis and LC–MS/MS for matrix-matched calibration purposes

BPs are commonly extracted by liquid–liquid extraction (LLE) and solid-phase extraction (SPE) either in cartridge format or dispersed (dSPE or QuEChERS) (Russo et al. 2019a). These sample preparation techniques have been established since at least 4 decades and they are based on the selected partitioning of the target analytes in a water immiscible organic solvent layer (typically n-hexane, ethyl acetate, or their mixtures) in LLE and on selective absorption/partitioning on a solid sorbent in (d) SPE. It should be noted that both LLE and (d) SPE tend to have an environmental impact particularly when the latter is operated in reversed phase. Newer, more selective and with reduced carbon footprint sample preparation approaches are Microwave-Assisted Extraction (MAE), Dispersive Liquid Micro-Liquid Extraction (DLLME), and molecularly imprinted solid-phase extraction (MISPE).

Due to the development of analytical columns of smaller i.d. (particularly for LC), liquid–liquid microextraction (LLME), DLLME, vortex-assisted liquid–liquid microextraction (VALLME), and single-drop microextraction (SDME) have been widely adopted in food analysis providing enhanced cost-effectiveness and increased recovery rates, with the use of only minimal solvent volumes. DLLME requires two-step process: first, the analyte is extracted and dispersed, and then, the resulting mixture undergoes centrifugation. It employees a ternary blend consisting of an extraction solvent, a dispersion solvent, and the aqueous sample; the choice of the extraction solvents is pivotal for a good recovery and selectivity. The limitations of DLLME in solvent selection have been overcome by VALLME, introduced by Psillakis (2019), being the method easier in operation, more cost-effective, and the additional dispersing solvents-free. QuEChERS is another option in food sample preparation employing extraction salts, followed by the clean-up of supernatant using dSPE. On the other hand, SPE separates analytes by capturing them between a solid phase (sorbent) and a liquid phase (sample). The method requires the column conditioning, sample loading, washing, and elution, with various options related to the chosen sorbents, formats (cartridges, disks, 96-well plates, and pipette tips), and whether automated (online SPE) or conventional (off-line SPE). SPE offers the possibility to simultaneously enrich trace compounds, eliminate matrix interferences providing high pre-concentration factors. This technique facilitates efficient pre-concentration, sample clean-up, and compatibility with diverse detection techniques, making it a preferred choice in modern food analytical chemistry. Furthermore, it has undergone significant improvements, such as dispersive solid-phase extraction (d-SPE), solid-phase microextraction (SPME), magnetic solid-phase extraction (MSPE), and molecularly imprinted polymers (MIP) extraction. The latter is a particular SPE based on molecular recognition principles. MIP sorbents possess recognition sites that match the shape and physicochemical features of the target analyte, known as the template molecule. Considering both GC and LC methods to identify and quantify BPA and/or its analogues, we summarized in Tables 1 and 2 all the studies gathered in the scientific literature from 2018 to 2024.

**Fig. 2:**
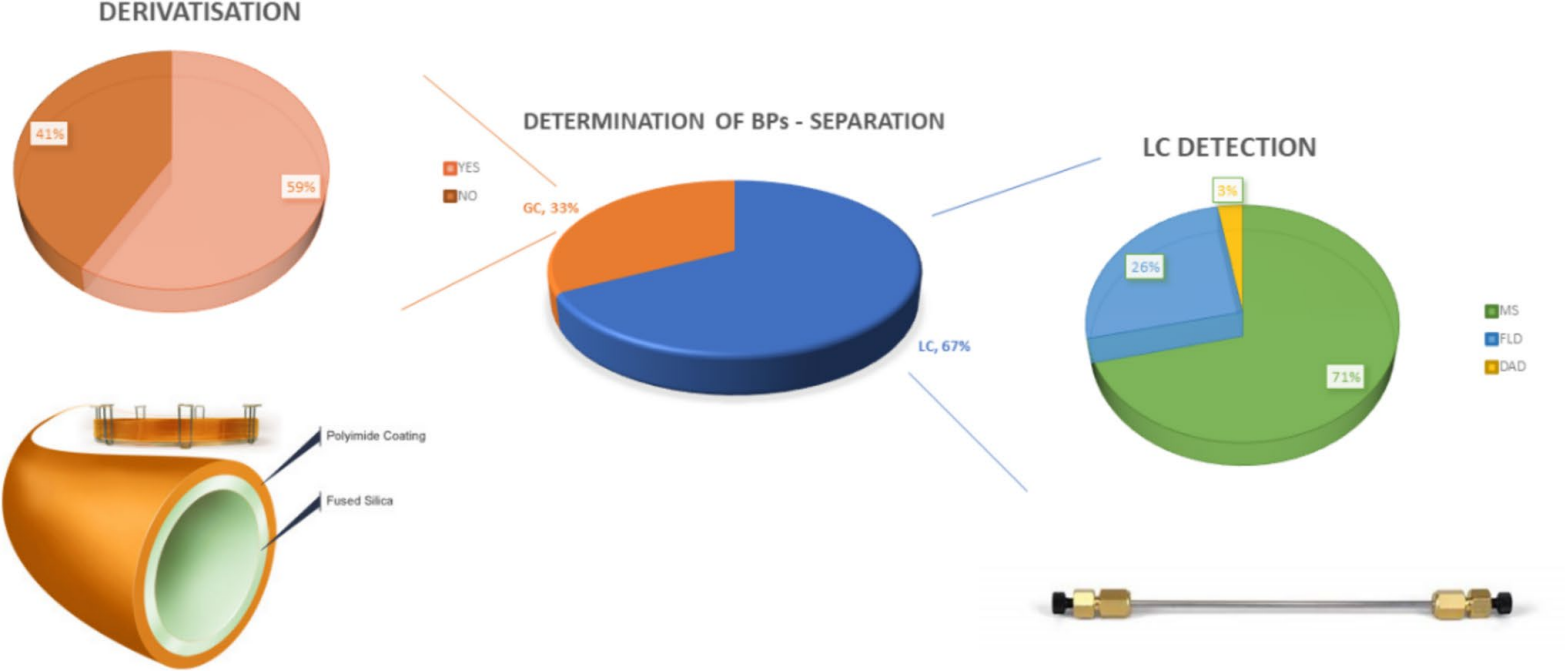
Percentages of the separation techniques used to determine BPA and its analogue from 2018 to date. In pie to pie: LC detection techniques in LC (right) and derivatisation (Y/N) in GC (left)

By examining Fig. 2, it is evident that LC is the most commonly applied analytical approach accounting for more than two-thirds of the sourced articles. This is not surprising as overall LC allows analysis of compounds over a wider range of polarity and molecular volume, which is not the case for GC for which molecules need to feature inherent volatility or otherwise be derivatised. Due to its moderate hydrophobicity, BPA is hereby determined in reversed phase and the selectivity that is more frequently exploited is certainly C18 as such or sometimes polar embedded, typically used to enhance the retention of the more hydrophilic analyte which is relevant when BPA and its derivatives are included in multiresidue analysis. Some research groups use commercially available C18 columns using sterically hindered silanes to protect the siloxane bond, allowing to operate the chromatographic experiment at a wider pH range (typically pH 2–10). Again, this is not relevant for BPA as its phenolic OH moieties have pKa values around 10 and these are predominantly undissociated at the experimental pH. However, it could be useful for improving the peak shape and resolution of basic substances, which may be relevant when BPA is analysed along with some pharmaceuticals, for instance. A good share of phenyl stationary phases can also be observed. This is a reasoned choice as such a stationary phase depicts the interactions occurring between π-systems, that are widely represented in bisphenols, and allow exploitation of distinctive selectivities particularly when operated with alcohols (such as methanol and ethanol) as organic modifiers. With these regards, phenyl-hexyl stationary phases provide usefulness as they combine both hydrophobic and π-stacking interactions that are particularly ideal for branched BPs. Another selectivity that is now emerging for BP analysis is pentafluorophenyl, which was introduced as it affords orthogonality when compared to alkyl bonded phases. PFP features different selectivity because of the original overlapping of π–π, dipole, hydrogen bonding, and ionic interactions.

In pure principle, GC, which was conducted in 33% of the examined articles, is the technique allowing the highest resolution and efficiency. Of note, GC–MS is typically a more cost-effective alternative when compared with LC–MS of identical resolution. Moreover, matrix effects are typically minimised by the intrinsic selectivity of the analytical technique that filters out polar interferents. In terms of the selectivity that are conventionally exploited in GC, these are typically nonpolar (5%-phenyl)-methylpolysiloxane capillary column (HP-5Ms) or its equivalent (5%-diphenyl)-dimethylpolysiloxane (DB-5Ms). The format, particularly film thickness and length, can be different depending on the applied temperature gradient, the matrix, and the carrier has employed. BPA can be also analysed without any derivatisation; however, this step is performed in the 59% of the scientific literature examined as it often provides sharper peaks and increased selectivity, particularly in complex mixtures. The most widely used derivatisation procedures are silylation and acetylation. The former is performed by reacting BPA and its derivatives with bis(trimethylsilyl)trifluoroacetamide (BSTFA) often with trimethylchlorosilane to favour the formation of a single derivatives which for BPA is trimethylsilyl BPA, [M-15]+, detectable at m/z 357 in electron impact mode (EI). The latter is achieved by reaction with acetic or trifluoroacetic anhydride leading to a base peak, [M-15]+, detectable at m/z 405 in EI due to the loss of a methyl group (Peñalver et al. 2021).

## Occurrence of BPs in food

The diet is the main exposure route for BPs, as these can migrate from FCM, even if food can also extract BPs during processing and transportation, especially when PVC gloves or tubing are used. Even storage containers and cookware can accidentally release BPs into food through inks, adhesives, or coatings. According to the available monitoring European research articles from 2018 until nowadays, we reported the BPs occurrence in various foodstuffs in Table 3 along with their concentration levels, even if, for some food categories such as meat and cereals, we found only few monitoring assessments and we therefore gathered them under the “mixed food” heading.

**Table 3:** Foodstuffs monitoring studies in the 2018–2023 time window

Country (year)	Matrix	Analytes	No. of samples	Min concentration	Max concentration	Ref
Spain (2018)	Fish, River Fish	BPA	59	< LOQ	59.09 ng g^−1^dw	Pico et al. (2019)
The Netherlands (2018)	canned food, beverages and drinkware	BPA + 16 6 BDGEs	22	< 0.07 ng/mL	68 ng g^−1^	van Leeuwen et al. (2019)
Spain (2018)	leafy and root vegetables	BPA	24	< LOQ	1.3 ng g^−1^	Aparicio et al. (2018)
Spain (2018)	Milk and Dairy, Infant formula	Tetrabromobisphenol (TBBPA) and BPA	50	0.77 ± 1.01 ng/mL free BPA 0.50 ± 0.02 ng/mL TBBPA	0.88 ± 1.01 ng mL^−1^ free BPA 0.58 ± 0.34 ng mL^−1^ TBBPA	Martinez et al. (2019)
Belgium (2018)	Freshwater	BPA	8	< LOQ	n.r	Huysman et al. (2019)
11 European Countries (2018)	Fish, Raw and Cooked Seafood	BPA	65	8.26 ng/g	69.1 ng g^−1^	Alvarez-Munoz et al. (2018)
Italy (2018)	Milk and Dairy, Raw Milk	BPA	22	< LOQ	2340 ng mL^−1^	Santonicola et al. (2018)
Italy (2018)	Milk and Dairy, Cow Milk	BPA	72	0.035 ng/mL	2.776 ng mL^−1^	Santonicola et al. (2019)
Spain (2018)	Vegetables, Lattuce	BPA and BPF	25	(0.59–2.32) 2.16 ng/g fw	199 ng g^−1^	Margenat et al. (2018)
Spain (2018)	Ready to eat baby food	BPA + 6	15	1.0 ng /g	49.2 ng g^−1^	Garcia-Corcoles et al. (2018)
Spain (2019)	Milk	BPA	50	£	£ 0.88 ng mL^−1^	Martinez et al. (2019)
Italy (2019)	Beverages	BPA + 6	52	> LOQ	1.358 ng mL^−1^	Russo et al. (2019b)
Italy (2019)	Beverages	BPA, BPB,BPF + BADGE + BFDGE	40	> LOQ	2.5 ng mL^−1^	Cirillo et al. (2019)
Spain (2019)	Fish	BPA	59	59.9 ng g^−1^	223.91 ng g^−1^	Pico et al. (2019)
Italy (2019)	Fruit Juices	BPA, BPB, BPF	46	0.50 ng mL^−1^	2.85 ng mL^−1^	Gallo et al. (2019)
Italy (2019)	Milk	BPA	72	0.035 ng mL^−1^	2.776 ng mL^−1^	Santonicola et al. (2019)
Czech Republic (2019)	Various (edible oils, butter, and chocolate)	BPA + 13	60	50 ng g^−1^	85 ng g^−1^	Vavrouš et al. (2019)
Greece (2020)	Canned food	BPA	6	7.3 ng g^−1^	42.3 ng g^−1^	Maragou et al. (2020)
Poland (2020)	Canned food	BADGE + 4; BFDGE + 2	20	1.6 ng g^−1^	693.6 ng g^−1^	Szczepańska et al. (2020)
Italy (2020)	Fish	BPA	44	< LOD	7.1 ng g^−1^	Di Marco Pisciottano et al. (2020)
Spain-Portugal (2020)	Canned vegetables	BPA	19	nd	nd	Vilarinho et al. (2020)
Italy, Algeria, Tunisia (2020)	Spices and aromatic herbs	BPA	53 26 65	< LOQ	< LOQ	Lo Turco et al. (2020)
Italy (2020)	Honey	BPA	6	< LOD	5.05 ng g^−1^	Notardonato et al. (2020)
Spain (2020)	Mixed	BPA + 8	40	0.17 ng g^−1^	33.19 ng g^−1^	González et al. (2020)
Portugal (2020)	Meat	BPA + 8	30	4.3 ng g^−1^	202.3 ng g^−1^	Cunha et al. (2020)
Spain (2020)	Cereal grain	BPA, BPF	16	1.6 ng g^−1^	1740 ng g^−1^	Albero et al. (2020)
North East Atlantic Ocean (2020)	Fish	BPA + 6	150	1.0 ng g^−1^	25.3 ng g^−1^	Barboza et al. (2020)
Spain (2020)	Cereal-based foodstuff	BPA	12	0.022 ng g^−1^	0.620 ng g^−1^	Azzouz et al. (2020)
Spain (2021)	Food and beverages	BPA + 18	10	0.071 ng g^−1^ 0.06 ng mL^−1^	84 ng g^−1^ 9.1 ng mL^−1^	Caballero-Casero and Rubio (2021)
Italy (2021)	Milk	BPF	84	< LOQ	2.686 ng mL^−1^	Santonicola et al. (2021a)
Italy (2021)	Milk	BPF	20	< LOQ	2.956 ng mL^−1^	Santonicola et al. (2021b)
Turkey (2022)	Fish	BADGE + 5 derivatives + BFDGE	33	60 ng g^−1^	220 ng g^−1^	Toptancı et al. (2022)
Italy (2022)	Plant based beverages	BPA, BPB, BPS	34	< LOD	18.17 ng mL^−1^	Schiano et al. (2022)
Italy (2022)	Drinking water, milk		108	0.01 ng mL^−1^ ( water), 0.1 ng ml^−1^ (milk)	1.0 ng mL^−1^ in water,2.0 ng mL^−1^ milk	Di Marco Pisciottano et al. (2022a)
Spain (2022)	Water and beverages	BPA, BPF, BPAF	20	nd	nd	Baute-Pérez et al. (2022)
Italy (2022)	Cheese Provola and olive cake samples	BPA + 8	20	1.69(provola) 3.27(olive cake) ng g^−1^	2.84/20.99 ng g^−1^	Liotta et al. (2022)
Italy (2022)	Milk	BPA + 19	46 + 15 with packaging	0.5 ng mL^−1^	5.6 ng mL^−1^	Di Marco Pisciottano et al. (2022b)
Portugal (2022)	Fish	BPA + 6	238	0.1 ng g^−1^	52.4 ng g^−1^	Petrarca et al. (2022b)
Poland (2022)	Milk	BPA + 6	19	0.12 ng mL^−1^	1.71 ng mL^−1^	Frankowski et al. (2022)
Portugal (2022)	seafood	BPA + 6	20	4.8 ng g^−1^	12.3 ng g^−1^	Petrarca et al. (2022a)
Turkey (2022)	Milk	BPA	11	2.1 ng mL^−1^	11.8 ng mL^−1^	Kartal Temel and Gürkan (2022)
France (2022)	MixedVegetables	BPA BADGE + 2 derivatives	12	43 ng g^−1^	1600 ng g^−1^	Cariou et al. (2022)
Turkey (2022)	Convenience foods (54 samples), canned vegetable oils (4 samples), olives (4 samples), and soft drinks (17 samples)	BPA BADGE + derivatives	79	nd	1056 ng g^−1^	Toptancı (2023)
Poland (2022)	Milk	BPA, BPS, BPF, BPAF, BPB, BPE	14	0.35 ng mL^−1^	0.87 ng mL^−1^	Frankowski et al. (2022)
Italy (2022)	Milk	BPA + 19	46	0.1 ng g^−1^	8.7 ng g^−1^	Di Marco Pisciottano et al. (2022b)
Switzerland (2023)	Canned food and beverages	BPA + 15	22	< LOD	40,650 ng g^−1^	Lucarini et al. (2023)

*nd* not detected

### Bisphenols in beverages

Several studies investigating BPs in mineral water and beverages were retrieved. Van Leeuwen et al. (2019) found trace levels of BPs with BPA being the most frequently detected in a limited selection of canned foods and beverages, along with BPS, BADGE, and some of its derivative (BADGE·HCl, BADGE·H_2_O, BADGE·2H_2_O) at relatively low concentration level. Di Marco et al*.* analysed over the total investigated 108 samples, 62 drinking water used in the milk production chain and the results showed very low concentrations (0.01–1.0 ng mL^−1^) of 17 investigated BPs (Di Marco Pisciottano et al. 2022a), while BPs were not found in the 20 bottled water analysed by Baute-Pérez et al. (2022). Only 14 samples of the 40 canned beers analysed by Cirillo et al. collected from the Italian market (Cirillo et al. 2019) were found to be contaminated by BPA or one of its analogues (concentrations ranging from 0.5 to 2.5 ng mL^−1^), while Russo et al. found that all of the 39 analysed canned beer but one single sample were positive for one or more BPs along with four energy drinks. BPA was found in 33% of the 46 fruit juice samples packaged in PET or Tetra Pak™ (0.50–2.85 ng mL^−1^) by Gallo et al. (2019). Detected levels of BPs in tonic water, tea, cola, and beer, along bottled water, were found to be ranging from 6.4 to 9.1 ng mL^−1^ in these investigated beverages marketed in Spain, according to a study authored by Caballero-Casero and Rubio (Caballero-Casero and Rubio 2021). Among beverages, also Italian plant-based beverages, such as soya, coconut, almond, oats, and rice, were found contaminated by some BPs with BPA occurring in 32% of the 34 samples screened (Schiano et al. 2022). Overall, the BP occurrence was attributed to various factors, such as the use of recycled PET, cross-contamination in bottling manufacturing, and even the occurrence of BPs in the water source itself. The BP levels lower (< LOQ) than previous years or absence in such liquid matrices, as reported in the Lucarini et al. research (Lucarini et al. 2023), may indicate the effectiveness of preventive measures implemented by the various governments, differently from the past. Since BPs are lipophilic compounds, they are often found in fatty and or oily foods, while trace levels are typically occurring in beverages. Various researchers looked into the presence of BPA in beverages and, generally, compared to other food categories, these were evaluated to contain relatively low concentrations of this chemical and/or of its analogues, but, on average, higher concentration levels were detected in tap waters as compared to bottled plastic water (Russo et al. 2022, 2019b).

The BPs concentration values examined from 2013 to 2018 in our previous review (Russo et al. 2019a), showed BPs concentration values in water, tap or bottled as low as 0.83–1.13 pg mL^−1^, broadly similar to those found in the scientific assessment considered in this updated piece of work (2018–2024) showing that an albeit minimal “baseline” contamination may occur due to environmental or packaging-related factors.

### Occurrence of bisphenols in fishes and seafood

More than 17% of global protein human consumption (Dawson et al. 2022) derives from the intake of seafood. Indeed, many edible fish contain various BPs analogues in their muscles and other tissues. Contamination can occur not only due to migration from packaging, but also to the presence of BPs in waterbodies. In fact, BPs were also found in river/sea fishes bought in local markets (Akhbarizadeh et al. 2021). Although both freshwater and seawater fish have been studied, most research articles focused on seafood with the most of the studies performed by countries, such as Spain and Portugal, overlooking the Atlantic Ocean. In general, BPs’ occurrence in this food category ranged from a minimum of 0.008 ngg^−1^ to a maximum 223.91 ng/ng^−1^. An interesting monitoring assessment was performed by Alvarez-Munoz et al. (Álvarez-Muñoz et al. 2018) who analyzed 65 different seafood samples among the 12 highly consumed fish and shellfish species from 11 European countries. They aimed to cover the seafood consumption habits in western, northern, and southern Europe. According to their study, the concentration levels spanned from 8.26 to 69.1 ng g^−1^. Based on BPA occurrence, the human exposure assessment indicated that the Spanish population has the highest BP exposure through seafood consumption. Indeed, Barboza et al. (Alvarez-Munoz et al. 2018) reported that the highest concentration of a BP was found in the liver (BPA, 302 ng g^−1^), while in the muscle (BPE), the levels were comparatively lower (BPE, 272 ng g^−1^). Considering the TDI of 4 μg kg^−1^ bw/ day for BPA assessed by the European Food Safety Authority (EFSA Panel on Food Contact Materials and Aids 2015), the estimated daily intake for BPs from fish consumption resulted higher than the recommended oral reference dose, posing a significant risk with potentially adverse effects on the human health over a lifetime. Petrarca and co-workers studied BP contamination in river fish and seafood by authoring two articles in the very same year focusing on river fishes (Petrarca et al. 2022b) and seafood (Petrarca et al. 2022a). The outcome was that BPA levels were higher in the river fishes (52.4 ng kg^−1^) than in seafood (23.1 ng kg^−1^). Considering our previous monitoring study on canned tuna and the published survey (2013–2017) (Russo et al. 2019a), the concentration values are, on average, rather similar or lower than in the past, with concentration values reaching values as high as 662 pg g^−1^ (Alabi et al. 2014). The global data analysis indicates that the presence of BPs in seafood can originate both from contamination occurring in the marine environment due to “seafood ready to eat” and canned seafood.

### Milk and dairy products

During the 2018–2021 period, the consumption of milk products within the European Union (EU-27) fluctuated, surging to 53.4 kg per capita in 2022, resulting in the EU-27 having one of the world's highest rates of per capita milk consumption. Even if several studies were carried out on milk and dairy products, these were unfortunately conducted mostly in Italy (9 versus 14), complicating the assessment of a wider European scenario. The highest concentration value (2.956 ng mL^−1^) of BPs in milk was found by Santonicola et al. (2021b), who developed an HPLC method for determining BPF levels during milk processing. In our previous review, we found that the highest BPs concentration in dairy was 800 μg kg^−1^ (Ferrer et al. 2011) in canned skimmed milk from Spanish market. Considering the high consumption of milk, especially by infants, this can be an important source of exposure to BPA and its analogues. However, the observed concentrations were below either the specific migration limit (SML) of 0.05 mg kg^−1^ into food from varnishes or coatings applied to food contact materials ((EU) 2018) or the t-TDI BPA values fixed. Nevertheless, BP traces can represent a risk to human health due to their potential synergistic effect with other EDCs.

### Mixed food

The most relevant study covering meat-based food was by Cunha et al. (2020) who investigated 30 canned meat samples (sausages, pâtés, and whole meals), gathered from Portugal markets but manufactured in three different countries (Portugal, Spain, and France). They found a total concentration sum of BPA and eight of its analogues, higher than 50 ng g^−1^, with a maximum of 236 ng g^−1^ in half of the analysed samples. As to cereals, we could retrieve only two studies (Albero et al. 2020; Azzouz et al. 2020). The former reported BPA in all investigated samples (barley, rice, wheat, and oat) at 1.6 to 1742 ng g^−1^ levels, and Bisphenol F at concentration levels up to 22 ng g^−1^ (6 samples). The latter covered the cereals rice, maize, and wheat, indicated concentration levels ranging from 0.022 pg g^−1^ to 0.620 ng g^−1^. The paucity of meat and cereals studies along with the limited number of analysed samples do not allow to draw any significant conclusion about this food category and, consequently, their impact on human health due to their consumption. In European Union regulations, composite foods are defined as those containing both processed animal and plant products, such as ready to eat baby food, leafy and root vegetables, and spices. We also report in Table 3 the assessment of BPs content in composite foods, even if, again, no conclusions can be drawn due to the extremely varying nature of the foodstuffs.

## Conclusions

The present review aimed at offering an update on the contamination levels of BPs in food and beverages in Europe from our latest review on the topic, which covered until the year 2017. Unfortunately, at this time, we could witness a scarcity of monitoring studies and a very homogeneous origin, with most country, if we except Poland, located in the southern part of Europe. For this reason, estimating a TDI would have been extremely challenging and would have offered a geographically sided view on this topic. This aspect may suggest that new plasticizers, hopefully with a reduced impact on the human health and/or mitigated endocrine-disrupting properties, are being developed and used in northern countries or that in these regions “greener” packaging such as glass and kraft cardboard are being exploited to a superior extent than in Southern Europe, reducing the amount of BP release or the likelihood of its occurrence. Another related possibility is that in the countries enforcing more stringent restrictions on the use of BPs, the contamination of foodstuff may indeed be so negligible to make such studies practically irrelevant and therefore discourage researchers to set up new monitoring work. However, considering that (1) the food and beverage market is extremely globalised, with frequent import and export of food commodities, (2) mixtures of contaminants, even at low concentration, can exert a synergistic effect on the endocrine system and (3) the levels found were not drastically lowered as compared to the past, it is still advisable to conduct monitoring studies to evaluate whether the enforcement of more stringent regulations translated into a decrease in the human exposure toBPs.

## Data Availability

Not applicable in this specific case as this is a review article.

## Abbreviations

CECs: Contaminants of Emerging Concern
PCPs: Personal Care Products
EDCs: Endocrine Disrupting Chemicals
BPA: Bisphenol A
BDGE: Bisphenol diglycidyl ether
FCM: Food Contact Material
BPs: Bisphenols
SPE: Solid-Phase Extraction
MIP: Molecular Imprinted Polymer
MAE-MISPE: Microwave-Assisted Extraction-Molecularly Imprinted Polymer-Solid Phase Extraction
FLD: Fluorescence Detection
MS: Mass Spectrometry
MSPD: Matrix Solid-Phase Dispersion
VA-DLLME: Vortex-Assisted-Dispersive Liquid Micro-Liquid Extraction
UAE-SPE: Ultrasound-Assisted Extraction
LLE: Liquid–Liquid Extraction
UVA-DLLME: Ultrasound-Vortex-Assisted Dispersive Liquid–Liquid Micro-Extraction
PC: Polycarbonate
PET: Polyethylene Terephthalate
TDI: Tolerated daily intake.
HDPE and LDPE: High‐ and Low‐Density Polyethylene
MC: Metallic Cans
PS: Polystyrene
PP: Polypropylene
PVC: Polyvinyl Chloride
SVHC: List of Substances of Very High Concern

## References

[CR1] Akhbarizadeh R, Russo G, Rossi S et al (2021) Emerging endocrine disruptors in two edible fish from the Persian Gulf: occurrence, congener profile, and human health risk assessment. Mar Pollut Bull 166:112241. 10.1016/J.Marpolbul.2021.112241 33711611 10.1016/J.Marpolbul.2021.112241

[CR2] Alabi A, Caballero-Casero N, Rubio S (2014) Quick and simple sample treatment for multiresidue analysis of bisphenols, bisphenol diglycidyl ethers and their derivatives in canned food prior to liquid chromatography and fluorescence detection. J Chromatogr A 1336:23–33. 10.1016/J.Chroma.2014.02.008 24594089 10.1016/J.Chroma.2014.02.008

[CR3] Albero B, Tadeo J, Pérez R (2020) Determination Of emerging contaminants in cereals by gas chromatography-tandem mass spectrometry. Front Chem 8:571668. 10.3389/Fchem.2020.571668 33195058 10.3389/Fchem.2020.571668 PMC7525029

[CR4] Ali M, Jaghbir M, Salam M, Al-Kadamany G, Damsees R, Al-Rawashdeh N (2018) Testing baby bottles for the presence of residual and migrated bisphenol A. Environ Monit Assess 191(1):7. 10.1007/S10661-018-7126-0 30535565 10.1007/S10661-018-7126-0

[CR5] Álvarez-Muñoz D, Rodríguez-Mozaz S, Jacobs S et al (2018) Pharmaceuticals and endocrine disruptors in raw and cooked seafood from European market: concentrations and human exposure levels. Environ Int 119:570–581. 10.1016/J.Envint.2018.07.006 30172197 10.1016/J.Envint.2018.07.006

[CR6] Álvarez-Muñoz D, Rodríguez-Mozaz S, Jacobs S et al (2018) Pharmaceuticals and endocrine disruptors in raw and cooked seafood from European market: concentrations and human exposure levels. Environ Int 119:570–581. 10.1016/J.Envint.2018.07.006 30172197 10.1016/J.Envint.2018.07.006

[CR7] Andra SS, Austin C, Yang J, Patel D, Arora M (2016) Recent advances in simultaneous analysis of bisphenol A and its conjugates in human matrices: exposure biomarker perspectives. Sci Total Environ 572:770–781. 10.1016/J.Scitotenv.2016.07.062 27586167 10.1016/J.Scitotenv.2016.07.062 PMC5099122

[CR8] Aparicio I, Martin J, Abril C, Jl S, Alonso E (2018) Determination of household and industrial chemicals, personal care products and hormones in leafy and root vegetables by liquid chromatography-tandem mass spectrometry. J Chromatogr A 1533:49–56. 10.1016/J.Chroma.2017.12.011 29229332 10.1016/J.Chroma.2017.12.011

[CR9] Astolfi M, Castellani F, Avino P et al (2021) Reusable water bottles: release of inorganic elements, phthalates, and bisphenol a in a “real use” simulation experiment. Separations 8(8):126 10.3390/separations8080126

[CR10] Azzouz A, Lp C, Hejji L, Ballesteros E (2020) Determination Of alkylphenols, phenylphenols, bisphenol A, parabens, organophosphorus pesticides and triclosan in different cereal-based foodstuffs by gas chromatography-mass spectrometry. Anal Bioanal Chem 412(11):2621–2631. 10.1007/S00216-020-02491-1 32055905 10.1007/S00216-020-02491-1

[CR11] Barboza L, Cunha SC, Monteiro C, Fernandes J, Guilhermino L (2020) Bisphenol A and its analogs in muscle and liver of fish from the north east atlantic ocean in relation to microplastic contamination exposure and risk to human consumers. J Hazard Mater 393:122419. 10.1016/J.Jhazmat.2020.122419 32155522 10.1016/J.Jhazmat.2020.122419

[CR12] Baute-Pérez D, Santana-Mayor Á, Av H-H, Socas-Rodríguez B, Má R-D (2022) analysis of alkylphenols, bisphenols and alkylphenol ethoxylates in microbial-fermented functional beverages and bottled water: optimization of a dispersive liquid-liquid microextraction protocol based on natural hydrophobic deep eutectic solvents. Food Chem 377:131921. 10.1016/J.Foodchem.2021.131921 34974406 10.1016/J.Foodchem.2021.131921

[CR13] Caballero-Casero N, Rubio S (2021) Comprehensive supramolecular solvent-based sample treatment platform for evaluation of combined exposure to mixtures of bisphenols and derivatives by liquid chromatography-tandem mass spectrometry. Anal Chim Acta 1144:14–25. 10.1016/J.Aca.2020.11.057 33453791 10.1016/J.Aca.2020.11.057

[CR14] Cañadas R, Garrido Gamarro E, Rm GM, Paniagua González G, Fernández Hernando P (2021) Occurrence of common plastic additives and contaminants in mussel samples: validation of analytical method based on matrix solid-phase dispersion. Food Chem 349:129169. 10.1016/J.Foodchem.2021.129169 33548886 10.1016/J.Foodchem.2021.129169

[CR15] Cao X-L, Zhao W, Dabeka R (2015) Di-(2-ethylhexyl) adipate and 20 phthalates in composite food samples from the 2013 Canadian Total Diet Study. Food Addit Contam Part A 32(11):1893–1901. 10.1080/19440049.2015.1079742 10.1080/19440049.2015.1079742 26359692

[CR16] Cariou R, Rivière M, Hutinet S et al (2022) Thorough investigation of non-volatile substances extractible from inner coatings of metallic cans and their occurrence in the canned vegetables. J Hazard Mater 435:129026. 10.1016/J.Jhazmat.2022.129026 35525007 10.1016/J.Jhazmat.2022.129026

[CR17] Chen D, Kannan K, Tan H et al (2016) Bisphenol analogues other than Bpa: environmental occurrence, human exposure and toxicity-a review. Environ Sci Technol 50(11):5438–5453. 10.1021/Acs.Est.5b05387 27143250 10.1021/Acs.Est.5b05387

[CR18] Cinelli G, Cuomo F, Ambrosone L, Venditti F, Lopez F (2019) Determination of bisphenol A in red wine using a double vortex–ultrasound-assisted microextraction assay: role of the interfacial properties. Biotechnol Prog 35(3):E2780. 10.1002/Btpr.2780 30697978 10.1002/Btpr.2780

[CR19] Cirillo T, Esposito F, Fasano E et al (2019) Bpa, Bpb, Bpf, badge and Bfdge in canned beers from the Italian market. Food Addit Contam Part B 12(4):268–274. 10.1080/19393210.2019.1650835 10.1080/19393210.2019.1650835 31412749

[CR20] Cunha S, Almeida C, Mendes E, Jo F (2011) Simultaneous determination of bisphenol A and bisphenol B in beverages and powdered infant formula by dispersive liquid-liquid micro-extraction and heart-cutting multidimensional gas chromatography-mass spectrometry. Food Addit Contam Part A 28(4):513–526. 10.1080/19440049.2010.542551 10.1080/19440049.2010.542551 21240700

[CR21] Cunha S, Inácio T, Almada M, Ferreira R, Jo F (2020) Gas chromatography-mass spectrometry analysis of nine bisphenols in canned meat products and human risk estimation. Food Res Int 135:109293. 10.1016/J.Foodres.2020.109293 32527484 10.1016/J.Foodres.2020.109293

[CR22] Dawson A, Li J, Kroon F (2022) Plastics for dinner: store-bought seafood, but not wild-caught from the great barrier reef, as a source of microplastics to human consumers. Environ Adv 8:100249. 10.1016/J.Envadv.2022.100249 10.1016/J.Envadv.2022.100249

[CR23] Dekant W, Völkel W (2008) Human exposure to bisphenol A by biomonitoring: methods, results and assessment of environmental exposures. Toxicol Appl Pharmacol 228(1):114–134. 10.1016/J.Taap.2007.12.008 18207480 10.1016/J.Taap.2007.12.008

[CR24] Di Bella G, Ben Mansour H, Ben Tekaya A et al (2018) Plasticizers and BPA residues in Tunisian and Italian culinary herbs and spices. J Food Sci 83(6):1769–1774. 10.1111/1750-3841.14171 29786850 10.1111/1750-3841.14171

[CR25] Di Marco Pisciottano I, Mita G, Gallo P (2020) Bisphenol A, octylphenols and nonylphenols in fish muscle determined by Lc/Esi-Ms/Ms after affinity chromatography clean up. Food Addit Contam Part B 13(2):139–147. 10.1080/19393210.2020.1740335 10.1080/19393210.2020.1740335 32208920

[CR26] Di Marco Pisciottano I, Albrizio S, Guadagnuolo G, Gallo P (2022a) Development and validation of a method for determination of 17 endocrine disrupting chemicals in milk, water, blood serum and feed by Uhplc-Ms/Ms. Food Addit Contam Part A Chem Anal Control Expo Risk Assess 39(10):1744–1758. 10.1080/19440049.2022.2104933 35947373 10.1080/19440049.2022.2104933

[CR27] Di Marco Pisciottano I, Guadagnuolo G, Busico F et al (2022b) Determination of 20 endocrine-disrupting compounds in the buffalo milk production chain and commercial bovine milk by Uhplc–Ms/Ms and Hplc-Fld. Animals 12(4):410 35203118 10.3390/ani12040410 PMC8868159

[CR28] Dueñas-Moreno J, Mora A, Kumar M, Meng X-Z, Mahlknecht J (2023) Worldwide risk assessment of phthalates and bisphenol a in humans: the need for updating guidelines. Environ Int 181:108294. 10.1016/J.Envint.2023.108294 37935082 10.1016/J.Envint.2023.108294

[CR29] Ehlert K, Beumer C, Groot M (2008) Migration of bisphenol a into water from polycarbonate baby bottles during microwave heating. Food Addit Contam Part A Chem Anal Control Expo Risk Assess 25(7):904–10. 10.1080/02652030701867867 18569009 10.1080/02652030701867867

[CR30] Fattore M, Russo G, Barbato F, Grumetto L, Albrizio S (2015) Monitoring of bisphenols in canned tuna from italian markets. Food Chem Toxicol 83:68–75. 10.1016/J.Fct.2015.05.010 26070504 10.1016/J.Fct.2015.05.010

[CR31] Ferrer E, Santoni E, Vittori S, Font G, Mañes J, Sagratini G (2011) Simultaneous determination of bisphenol A, octylphenol, and nonylphenol by pressurised liquid extraction and liquid chromatography-tandem mass spectrometry in powdered milk and infant formulas. Food Chem 126(1):360–367. 10.1016/J.Foodchem.2010.10.098 10.1016/J.Foodchem.2010.10.098

[CR32] Frankowski R, Grześkowiak T, Czarczyńska-Goślińska B, Zgoła-Grześkowiak A (2022) Occurrence and dietary risk of bisphenols and parabens in raw and processed cow’s milk. Food Addit Contam Part A 39(1):116–129. 10.1080/19440049.2021.1986234 10.1080/19440049.2021.1986234 34702142

[CR33] Gallo P, Di Marco PI, Esposito F et al (2017) Determination of Bpa, Bpb, Bpf, badge and BFDGE in canned energy drinks by molecularly imprinted polymer cleaning up and Uplc with fluorescence detection. Food Chem 220:406–412. 10.1016/J.Foodchem.2016.10.005 27855918 10.1016/J.Foodchem.2016.10.005

[CR34] Gallo P, Di Marco PI, Fattore M, Mg R, Seccia S, Albrizio S (2019) A method to determine bpa, bpb, and bpf levels in fruit juices by liquid chromatography coupled to tandem mass spectrometry. Food Addit Contam Part A 36(12):1871–1881. 10.1080/19440049.2019.1657967 10.1080/19440049.2019.1657967 31490737

[CR35] Gálvez-Ontiveros Y, Moscoso-Ruiz I, Rodrigo L, Aguilera M, Rivas A, Zafra-Gómez A (2021) Presence of parabens and bisphenols in food commonly consumed in Spain. Foods 10(1):92 33466450 10.3390/foods10010092 PMC7824906

[CR36] Garcia-Corcoles M, Cipa M, Rodriguez-Gomez R et al (2018) Determination of bisphenols with estrogenic activity in plastic packaged baby food samples using solid-liquid extraction and clean-up with dispersive sorbents followed by gas chromatography tandem mass spectrometry analysis. Talanta 178:441–448. 10.1016/J.Talanta.2017.09.067 29136846 10.1016/J.Talanta.2017.09.067

[CR37] González N, Cunha S, Ferreira R et al (2020) Concentrations of nine bisphenol analogues in food purchased from Catalonia (Spain): comparison of canned and non-canned foodstuffs. Food Chem Toxicol 136:110992. 10.1016/J.Fct.2019.110992 31760075 10.1016/J.Fct.2019.110992

[CR38] Guart A, Bono-Blay F, Borrell A, Lacorte S (2011) Migration of plasticizers phthalates, bisphenol A and alkylphenols from plastic containers and evaluation of risk. Food Addit Contam Part A Chem Anal Control Expo Risk Assess 28(5):676–685. 10.1080/19440049.2011.555845 21400322 10.1080/19440049.2011.555845

[CR39] Hananeh W, Al Rukibat R, Jaradat S, Borhan Al-Zghoul M (2021) Exposure assessment of bisphenol A by drinking coffee from plastic cups. Rocz Panstw Zakl Hig 72(1):49–53. 10.32394/Rpzh.2021.0146 33882786 10.32394/Rpzh.2021.0146

[CR40] Hejji L, Azzouz A, Lp C, Souhail B, Ballesteros E (2021) A multi-residue method for determining twenty-four endocrine disrupting chemicals in vegetables and fruits using ultrasound-assisted solid-liquid extraction and continuous solid-phase extraction. Chemosphere 263:128158. 10.1016/J.Chemosphere.2020.128158 33297136 10.1016/J.Chemosphere.2020.128158

[CR41] Herrero L, Quintanilla-López J, Fernández M, Gómara B (2021) Plasticisers and preservatives in commercial milk products: a comprehensive study on packages used in the Spanish market. Food Chem 338:128031. 10.1016/J.Foodchem.2020.128031 32950007 10.1016/J.Foodchem.2020.128031

[CR42] Huysman S, Van Meulebroek L, Janssens O et al (2019) Targeted quantification and untargeted screening of alkylphenols, bisphenol A and phthalates in aquatic matrices using ultra-high-performance liquid chromatography coupled to hybrid Q-Orbitrap mass spectrometry. Anal Chim Acta 1049:141–151. 10.1016/J.Aca.2018.10.045 30612645 10.1016/J.Aca.2018.10.045

[CR43] Jakimska A, Huerta B, Bargańska Ż, Kot-Wasik A, Rodríguez-Mozaz S, Barceló D (2013) Development of a liquid chromatography-tandem mass spectrometry procedure for determination of endocrine disrupting compounds in fish from Mediterranean rivers. J Chromatogr A 1306:44–58. 10.1016/J.Chroma.2013.07.050 23890552 10.1016/J.Chroma.2013.07.050

[CR44] Jensen T, Mustieles V, Bleses D et al (2019) Prenatal bisphenol a exposure is associated with language development but not with Adhd-related behavior in toddlers from the odense child cohort. Environ Res 170:398–405. 10.1016/J.Envres.2018.12.055 30623887 10.1016/J.Envres.2018.12.055

[CR45] Kanlayaprasit S, Thongkorn S, Panjabud P et al (2021) Autism-related transcription factors underlying the sex-specific effects of prenatal bisphenol A exposure on transcriptome-interactome profiles in the offspring prefrontal cortex. Int J Mol Sci 22(24):13201 34947998 10.3390/ijms222413201 PMC8708761

[CR46] Kartal Temel N, Gürkan R (2022) An indirect method for the analysis of bisphenol A, As A Mn(Iii)-chelate complex, in milk samples by ultrasound assisted-cloud point extraction/flame atomic absorption spectrometry. Anal Methods 14(26):2596–2607. 10.1039/D2ay00301e 35726781 10.1039/D2ay00301e

[CR47] Kawamura Y, Inoue K, Nakazawa H, Yamada T, Maitani T (2001) Cause of bisphenol a migration from cans for drinks and assessment of improved cans. Shokuhin Eiseigaku Zasshi 42(1):13–17 11383151 10.3358/shokueishi.42.13

[CR48] Krivohlavek A, Mikulec N, Budeč M et al (2023) Migration of Bpa from food packaging and household products on the Croatian market. Int J Environ Res Public Health 20(4):2877. 10.3390/Ijerph20042877 36833573 10.3390/Ijerph20042877 PMC9957217

[CR49] Kumar A, Singh D, Bhandari R, Ak M, Kaur S, Singh B (2023) Bisphenol A in canned soft drinks, plastic-bottled water, and household water tank from Punjab, India. J Hazard Mater Adv 9:100205. 10.1016/J.Hazadv.2022.100205 10.1016/J.Hazadv.2022.100205

[CR50] Lapviboonsuk J, Leepipatpiboon N (2014) A simple method for the determination of bisphenol A diglycidyl ether and its derivatives in canned fish. Anal Methods 6(15):5666–5672. 10.1039/C4ay00757c 10.1039/C4ay00757c

[CR51] Lestido-Cardama A, Vázquez Loureiro P, Sendón R, Paseiro Losada P, Rodríguez Bernaldo De Quirós A (2021) Application of chromatographic analysis for detecting components from polymeric can coatings and further determination in beverage samples. J Chromatogr A 1638:461886. 10.1016/J.Chroma.2021.461886 33465586 10.1016/J.Chroma.2021.461886

[CR52] Liao C, Kannan K (2012) Determination of free and conjugated forms of bisphenol a in human urine and serum by liquid chromatography-tandem mass spectrometry. Environ Sci Technol 46(9):5003–5009. 10.1021/Es300115a 22489688 10.1021/Es300115a

[CR53] Lim D, Kwack S, Kim K, Kim H, Lee B (2009) Potential risk of bisphenol a migration from polycarbonate containers after heating, boiling, and microwaving. J Toxicol Environ Health A 72(21–22):1285–91. 10.1080/15287390903212329 20077198 10.1080/15287390903212329

[CR54] Liotta L, Litrenta F, Lo Turco V et al (2022) Evaluation of chemical contaminants in conventional and unconventional ragusana provola cheese. Foods 11(23):3817 36496625 10.3390/foods11233817 PMC9740842

[CR55] Lo Turco V, Ag P, Ben Mansour H, Dugo G, Di Bella G (2020) Plasticizers and bpa in spices and aromatic herbs of Mediterranean areas. Nat Prod Res 34(1):87–92. 10.1080/14786419.2019.1591403 30905174 10.1080/14786419.2019.1591403

[CR56] Lucarini F, Gasco R, Staedler D (2023) Simultaneous quantification of 16 bisphenol analogues in food matrices. Toxics 11(8):665. 10.3390/Toxics11080665 37624170 10.3390/Toxics11080665 PMC10458576

[CR57] Maragou N, Thomaidis N, Theodoridis G, Lampi E, Koupparis M (2020) Determination of bisphenol A in canned food by microwave assisted extraction, molecularly imprinted polymer-solid phase extraction and liquid chromatography-mass spectrometry. J Chromatogr B 1137:121938. 10.1016/J.Jchromb.2019.121938 10.1016/J.Jchromb.2019.121938 31881513

[CR58] Margenat A, Matamoros V, Diez S, Canameras N, Comas J, Jm B (2018) Occurrence and bioaccumulation of chemical contaminants in lettuce grown in peri-urban horticulture. Sci Total Environ 637–638:1166–1174. 10.1016/J.Scitotenv.2018.05.035 29801210 10.1016/J.Scitotenv.2018.05.035

[CR59] Martinez M, Castro I, Rovira J et al (2019) Early-life intake of major trace elements, bisphenol a, tetrabromobisphenol a and fatty acids: comparing human milk and commercial infant formulas. Environ Res 169:246–255. 10.1016/J.Envres.2018.11.017 30476748 10.1016/J.Envres.2018.11.017

[CR60] Mercogliano R, Santonicola S, Albrizio S, Ferrante M (2021) Occurrence of bisphenol a in the milk chain: a monitoring model for risk assessment at a dairy company. J Dairy Sci 104(5):5125–5132. 10.3168/Jds.2020-19365 33685697 10.3168/Jds.2020-19365

[CR61] Michałowicz J (2014) Bisphenol A-sources, toxicity and biotransformation. Environ Toxicol Pharmacol 37(2):738–758. 10.1016/J.Etap.2014.02.003 24632011 10.1016/J.Etap.2014.02.003

[CR62] Molina-López A, Bujalance-Reyes F, Ayala-Soldado N, Mora-Medina R, Lora-Benítez A, Moyano-Salvago R (2023) An overview of the health effects of bisphenol a from a one health perspective. Animals 13(15):2439 37570248 10.3390/ani13152439 PMC10417040

[CR63] Noonan G, Ackerman L, Begley T (2011) Concentration of bisphenol a in highly consumed canned foods on the U.S. market. J Agric Food Chem 59(13):7178–7185. 10.1021/Jf201076f 21598963 10.1021/Jf201076f

[CR64] Notardonato I, Passarella S, Ianiri G, Di Fiore C, Russo M, Avino P (2020) Analytical scheme for simultaneous determination of phthalates and bisphenol a in honey samples based on dispersive liquid-liquid microextraction followed by Gc-It/Ms. Effect of the thermal stress on Pae/Bp-A levels. Methods Protoc 3:23 32213842 10.3390/mps3010023 PMC7189663

[CR65] Peñalver R, Arroyo-Manzanares N, Campillo N, Viñas P (2021) Targeted and untargeted gas chromatography-mass spectrometry analysis of honey samples for determination of migrants from plastic packages. Food Chem 334:127547. 10.1016/J.Foodchem.2020.127547 32693334 10.1016/J.Foodchem.2020.127547

[CR66] Petraccia L, Liberati G, Masciullo S, Grassi M, Fraioli A (2006) Water, mineral waters and health. Clin Nutr 25(3):377–385. 10.1016/J.Clnu.2005.10.002 16314004 10.1016/J.Clnu.2005.10.002

[CR67] Petrarca M, Fernandes J, Marmelo I, Marques A, Cunha S (2022a) Multi-analyte gas chromatography-mass spectrometry method to monitor bisphenols, musk fragrances, ultraviolet filters, and pesticide residues in seafood. J Chromatogr A 1663:462755. 10.1016/J.Chroma.2021.462755 34968957 10.1016/J.Chroma.2021.462755

[CR68] Petrarca M, Menezes-Sousa D, Ferreira R et al (2022b) Occurrence and risk assessment of endocrine-disrupting compounds in fish muscle: the case study of the douro river estuary (North East Atlantic Ocean). Environ Res 215:114236. 10.1016/J.Envres.2022.114236 36058278 10.1016/J.Envres.2022.114236

[CR69] Pico Y, Belenguer V, Corcellas C et al (2019) Contaminants of emerging concern in freshwater fish from four Spanish rivers. Sci Total Environ 659:1186–1198. 10.1016/J.Scitotenv.2018.12.366 31096332 10.1016/J.Scitotenv.2018.12.366

[CR70] Polson C, Sarkar P, Incledon B, Raguvaran V, Grant R (2003) Optimization of protein precipitation based upon effectiveness of protein removal and ionization effect in liquid chromatography-tandem mass spectrometry. J Chromatogr B Analyt Technol Biomed Life Sci 785(2):263–275. 10.1016/S1570-0232(02)00914-5 12554139 10.1016/S1570-0232(02)00914-5

[CR71] Psillakis E (2019) Vortex-assisted liquid-liquid microextraction revisited. TrAC Trends Anal Chem 113:332–339. 10.1016/J.Trac.2018.11.007 10.1016/J.Trac.2018.11.007

[CR72] Ramírez V, Merkel S, Tietz T, Rivas A (2023) Risk assessment of food contact materials. EFSA J 21(S1):E211015. 10.2903/J.Efsa.2023.E211015 38047134 10.2903/J.Efsa.2023.E211015 PMC10687752

[CR73] Rochester JR (2013) Bisphenol A and human health: a review of the literature. Reprod Toxicol 42:132–155. 10.1016/J.Reprotox.2013.08.008 23994667 10.1016/J.Reprotox.2013.08.008

[CR74] Rosenmai A, Dybdahl M, Pedersen M et al (2014) Are structural analogues to bisphenol a safe alternatives? Toxicol Sci 139(1):35–47. 10.1093/Toxsci/Kfu030 24563381 10.1093/Toxsci/Kfu030

[CR75] Russo G, Barbato F, Cardone E, Fattore M, Albrizio S, Grumetto L (2018) Bisphenol A and bisphenol S release in milk under household conditions from baby bottles marketed in Italy. J Environ Sci Health B 53(2):116–120. 10.1080/03601234.2017.1388662 29172986 10.1080/03601234.2017.1388662

[CR76] Russo G, Barbato F, Mita D, Grumetto L (2019a) Occurrence of bisphenol A and its analogues in some foodstuff marketed in Europe. Food Chem Toxicol 131:110575. 10.1016/J.Fct.2019.110575 31201899 10.1016/J.Fct.2019.110575

[CR77] Russo G, Varriale F, Barbato F, Grumetto L (2019b) Are canned beverages industries progressively switching to bisphenol Af? J Food Sci 84(11):3303–3311. 10.1111/1750-3841.14833 31671224 10.1111/1750-3841.14833

[CR78] Russo G, Laneri S, Di Lorenzo R et al (2022) Monitoring of pollutants content in bottled and tap drinking water in Italy. Molecules 27(13):3990. 10.3390/Molecules27133990 35807230 10.3390/Molecules27133990 PMC9268051

[CR79] Santonicola S, Ferrante M, Leo G, Murru N, Anastasio A, Mercogliano R (2018) Study on endocrine disruptors levels in raw milk from cow’s farms: risk assessment. Ital J Food Saf 7(3):7668. 10.4081/Ijfs.2018.7668 30538962 10.4081/Ijfs.2018.7668 PMC6240925

[CR80] Santonicola S, Mc F, Murru N, Gallo P, Mercogliano R (2019) Hot topic: bisphenol a in cow milk and dietary exposure at the farm level. J Dairy Sci 102(2):1007–1013. 10.3168/Jds.2018-15338 30594366 10.3168/Jds.2018-15338

[CR81] Santonicola S, Albrizio S, Mc F, Raffaelina M (2021a) Study on bisphenol F, a bisphenol A analogue, at a dairy company: health hazard and risk assessment. Food Chem Toxicol 154:112334. 10.1016/J.Fct.2021.112334 34118346 10.1016/J.Fct.2021.112334

[CR82] Santonicola S, Ferrante M, Colavita G, Mercogliano R (2021b) Development of a high-performance liquid chromatography method to assess bisphenol F levels in milk. Ital J Food Saf 10(4):9975. 10.4081/Ijfs.2021.9975 35036367 10.4081/Ijfs.2021.9975 PMC8696387

[CR83] Schiano M, Sodano F, Cassiano C et al (2022) Quantitative determination of bisphenol a and its congeners in plant-based beverages by liquid chromatography coupled to tandem mass spectrometry. Foods 11:3853 36496660 10.3390/foods11233853 PMC9737382

[CR84] Serra H, Beausoleil C, Habert R, Minier C, Picard-Hagen N, Michel C (2019) Evidence for bisphenol b endocrine properties: scientific and regulatory perspectives. Environ Health Perspect 127(10):106001. 10.1289/Ehp5200 31617754 10.1289/Ehp5200 PMC6867436

[CR85] Sirot V, Rivière G, Leconte S et al (2021) Infant total diet study in france: exposure to substances migrating from food contact materials. Environ Int 149:106393. 10.1016/J.Envint.2021.106393 33529853 10.1016/J.Envint.2021.106393

[CR86] Szczepańska N, Kubica P, Płotka-Wasylka J, Kudłak B, Namieśnik J (2020) Ultrasound assisted solvent extraction of porous membrane-packed samples followed by liquid chromatography-tandem mass spectrometry for determination of badge, Bfdge and their derivatives in packed vegetables. Sci Total Environ 708:135178. 10.1016/J.Scitotenv.2019.135178 31791752 10.1016/J.Scitotenv.2019.135178

[CR87] Toptancı İ (2023) Risk assessment of bisphenol related compounds in canned convenience foods, olives, olive oil, and canned soft drinks In Turkey. Environ Sci Pollut Res Int 30(18):54177–54192. 10.1007/S11356-023-26228-6 36869959 10.1007/S11356-023-26228-6

[CR88] Toptancı İ, Kıralan M, Ketenoglu O, Mf R (2022) Monitoring of bisphenol a diglycidyl ether (badge) and some derivatives in fish products in the Turkey market. Environ Sci Pollut Res 29(35):52788–52795. 10.1007/S11356-022-19587-Z 10.1007/S11356-022-19587-Z 35267165

[CR89] Usman A, Ahmad M (2016) From Bpa to its analogues: is it a safe journey? Chemosphere 158:131–142. 10.1016/J.Chemosphere.2016.05.070 27262103 10.1016/J.Chemosphere.2016.05.070

[CR90] Van Leeuwen S, Bovee T, Awchi M et al (2019) Bpa, badge and analogues: a new multi-analyte Lc-Esi-Ms/Ms method for their determination and their in vitro (anti)estrogenic and (anti)androgenic properties. Chemosphere 221:246–253. 10.1016/J.Chemosphere.2018.12.189 30640007 10.1016/J.Chemosphere.2018.12.189

[CR91] Vandenberg L, Hauser R, Marcus M, Olea N, Wv W (2007) Human exposure to bisphenol A (Bpa). Reprod Toxicol 24(2):139–177. 10.1016/J.Reprotox.2007.07.010 17825522 10.1016/J.Reprotox.2007.07.010

[CR92] Vavrouš A, Ševčík V, Dvořáková M, Čabala R, Moulisová A, Vrbík K (2019) Easy and inexpensive method for multiclass analysis of 41 food contact related contaminants in fatty food by liquid chromatography-tandem mass spectrometry. J Agric Food Chem 67(39):10968–10976. 10.1021/Acs.Jafc.9b02544 31487165 10.1021/Acs.Jafc.9b02544

[CR93] Vilarinho F, Lestido-Cardama A, Sendón R, Rodríguez Bernaldo De Quirós A, Vaz M, Sanches-Silva A (2020) Hplc with fluorescence detection for determination of bisphenol a in canned vegetables: optimization, validation and application to samples from Portuguese and Spanish markets. Coatings 10:624 10.3390/coatings10070624

[CR94] Vom Saal FS, Hughes C (2005) An extensive new literature concerning low-dose effects of bisphenol a shows the need for a new risk assessment. Environ Health Perspect 113(8):926–933. 10.1289/Ehp.7713 16079060 10.1289/Ehp.7713 PMC1280330

[CR95] Xiong L, Yan P, Chu M, Gao Y, Li W, Yang X (2018) A rapid and simple Hplc-Fld screening method with quechers as the sample treatment for the simultaneous monitoring of nine bisphenols in milk. Food Chem 244:371–377. 10.1016/J.Foodchem.2017.10.030 29120796 10.1016/J.Foodchem.2017.10.030

[CR96] Agencies Ec (2023) https://echa.europa.eu/substance-information/-/substanceinfo/100.001.133 Accessed 13 May 2024

[CR97] Efsa Panel On Food Contact Materials E, Flavourings, Aids P (2015) Scientific opinion on the risks to public health related to the presence of bisphenol A (Bpa) in foodstuffs. Efsa J 13(1):3978 10.2903/J.Efsa.2015.3978

[CR98] (Eu) Cr (2005) On the restriction of use of certain epoxy derivatives in materials and articles intended to come into contact with food. Vol No 1895/2005, Official Journal Of The European Union

[CR99] (Eu) Cr (2011) On plastic materials and articles intended to come into contact with food. Vol No 10/2011 Official Journal Of The European Union

[CR100] (Eu) Cr (2018) On the use of bisphenol a in varnishes and coatings intended to come into contact with food and Amending Regulation (Eu) No 10/2011 As Regards The Use Of That Substance In Plastic Food Contact Materials. Vol No 2018/213, Official Journal Of The European Union

[CR101] Federal Law Gazette Part II (2011) Vol No.327/2011

[CR102] Group assessment of bisphenols identifies need for restriction (2022). https://echa.europa.eu/-/group-assessment-of-bisphenols-identifies-need-for-restriction Accessed 13 May 2024 Echa/Nr/22/08

[CR103] Agency Tdep (2022) https://eng.mst.dk/chemicals/chemicals-in-products/chemical-legislation/danish-action-plans-on-chemicals Accessed 13 May 2024

